# Supplementary material for: An upstream secondary DNA motif within the IL3 insulator CTCF binding site is required for enhancer-blocking insulator activity
Source: PLoS One. 2026 Feb 9;21(2):e0340247. doi: 10.1371/journal.pone.0340247 (PMC12885311; doi:10.1371/journal.pone.0340247)
Supplement: S1 File — (PDF) [file pone.0340247.s001.pdf]

Supporting information Bowers and Cockerill

An upstream secondary DNA motif within the IL3 insulator CTCF binding site is required for enhancer-blocking insulator activity.

Sarion R. Bowers and Peter N. Cockerill

S1 figure **CTCF sites at me3K27H3 chromatin boundaries**

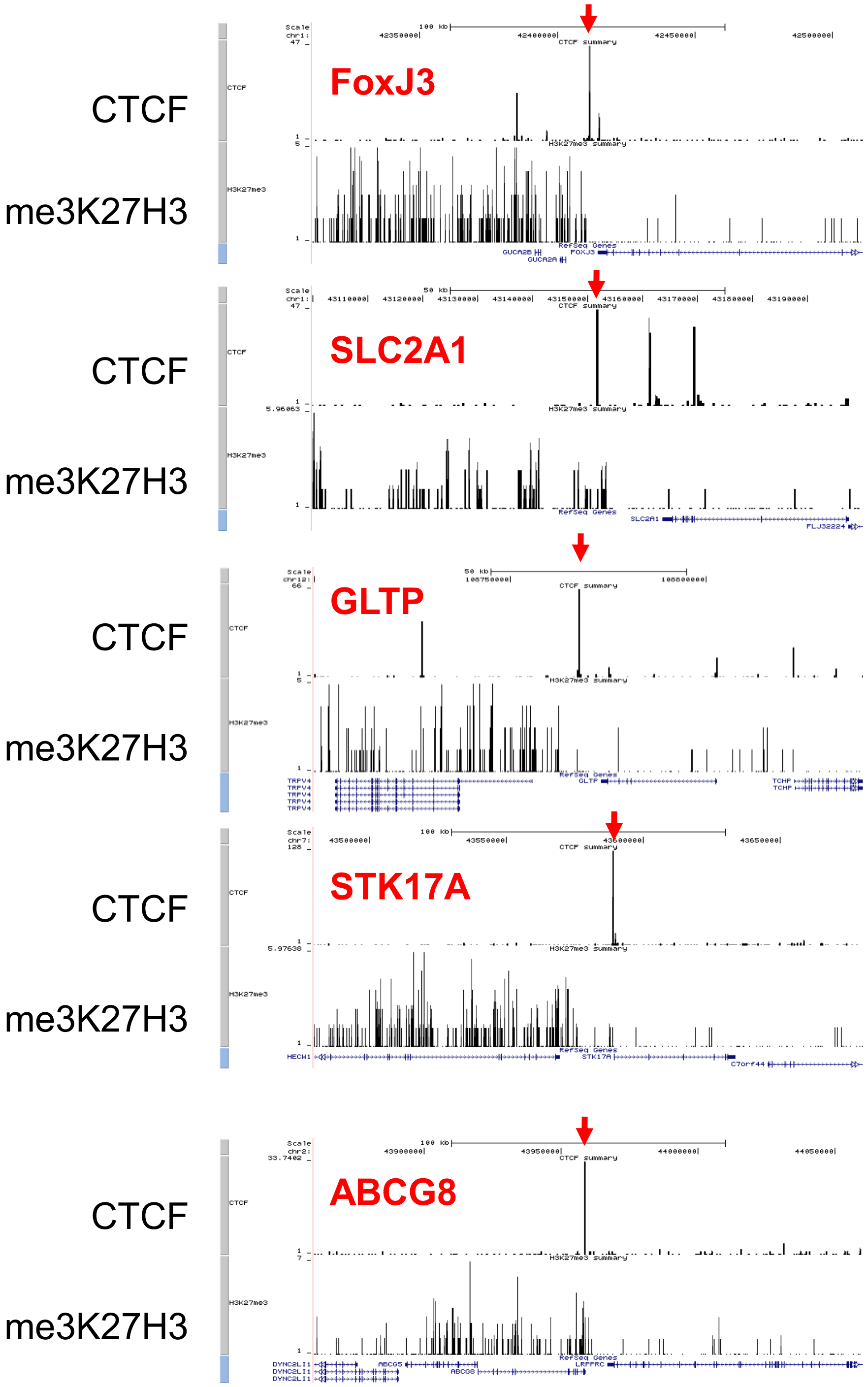

S1 Figure. Locations of CTCF sites at histone H3K27me27 chromatin boundaries.
Data was extracted from a UCSC genome browser session for which the authors had uploaded their published histone H3K27me27 and CTCF ChIP data. This session was access via a link in the authors publication: https://dir.nhlbi.nih.gov/papers/lmi/epigenomes/hgtcell.aspx [24, 47].

S2 figure Unmanipulated original images used in figure 2

The boxed regions of panel C indicate irrelevant lanes that were removed before preparing Fig 1C.

Fig 2C original image with deleted lanes marked by boxes

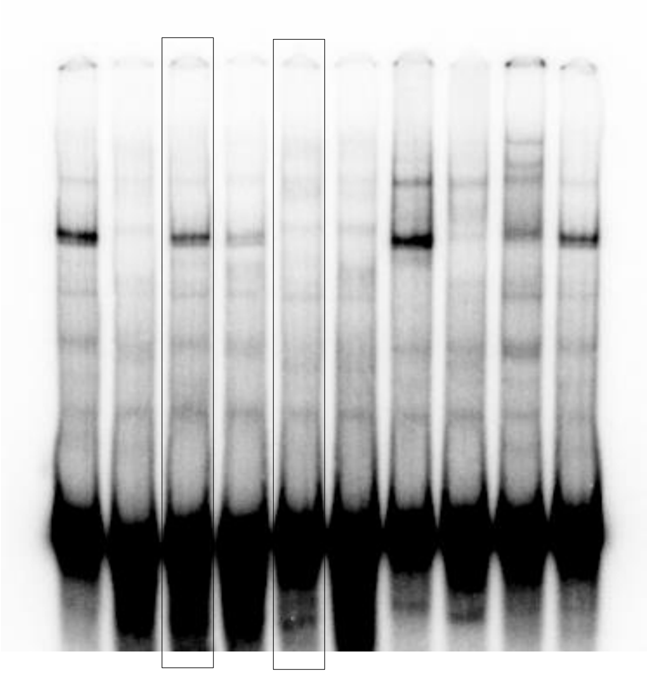

Fig 2E original image

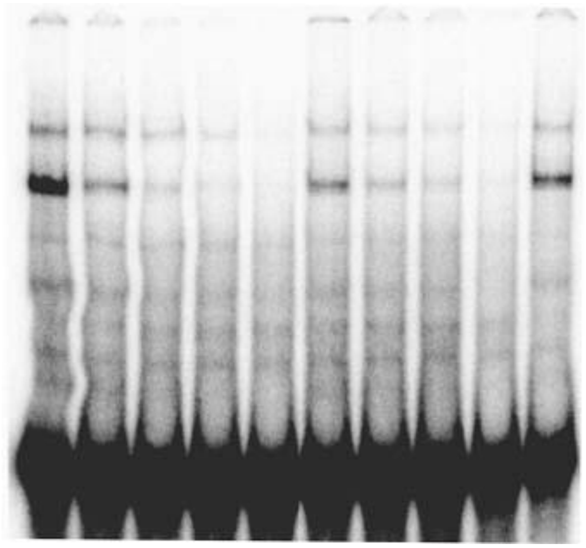

Fig 2F original image

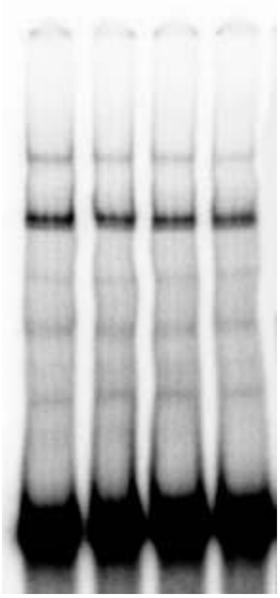

S3 Table

CTCF sites in the IL-3 gene insulator

```
                       2° upstream          1° core
                          motif              motif
Global consensus       CTGCAGTTCCNNNNNTGGCCACCAGGGGGCGCC
of composite sites     G  TTACGTT     C     G   AT   AGT
                            C
```

IL-3 +2.9 kb

```
HUMAN       AGGGGCGGCGGCTGCAGTTTCTGGAAGGACCACTAGGGGGAGACATGC
ORANGUTAN   ......................................A.........
RHESUS      .........T..............................G...G...
BABOON      .........T..................................G...
MOUSE LEMUR .....AC..T.....G.Gg..C..C......t.GG....AG.cGTGAG
BUSHBABY    ....AGT..T................t....t....a......G....
TREE SHREW  T....AT..A.........c......A............A..g..G..
MOUSE       TA...ACA.T.........ctAA.T.t...............g...T.
RAT         TA.A.GCA.T.........ctAA.T.t..........A..GAg...T.
GUINEA PIG  T..A.AT..TA............A..t.............cAgtG.C.
RABBIT      GAAA..T..T.....t  ..........A.........a.....G...T.
HORSE       T.A..AT..T...........CA.TGtAG.............g.....
DOG         .A.A.AA..TC.............T..A.............Ag..G..
ELEPHANT    TA..AACCTT.g..t.........T...G...................
ROCK HYRAX  ---------A.g............T...G..t...A..........A.
ARMADILLO   GA...AA..T.............TC.A.G....C.C...A..g.....
SLOTH       TA...AT..T.............TCT..G....C........g.....
```

IL-3 +4.2 kb

```
HUMAN       AGAGGATGCTGCTGCAGTTTCTGGATTGACCACTAGGGGGAGGTGTGT
ORANGUTAN   ....................................a...........
RHESUS      .......................A........g...............
MARMOSET    ......G..............C..............a...........
MOUSE LEMUR TAG...CA...........c....TGG......c......c....CCG
BUSHBABY    TAG..CCTG..........c..TCTAA......c.............C
TREESHREW   T.G..C..TG.A.........CA..AG......a...A..c......C
MOUSE       CAGA.T.CT.T........cG..A.A..........a..A..AAAATG
RAT         .TGA..CAT.C........ct..A.A..........a..Ac.A.A..C
GUINEA PIG  CT........Ag...t......AACA.......c..a.A...c....C
ALPACA      CAG................c..T..GGAG..........AG.cACGCC
HORSE       T....................CA.TG.AG..............CA..C
CAT         T.GA..A.................TGGAG..........C...CAG.C
DOG         .AGA..A...C.............TAGA.............a.CCACC
```

Human IL-3 +2.9/+4.2 identities

```
            AG*GG**GC*GCTGCAGTTTCTGGA**GACCACTAGGGGGAG***TG*
```

S3 Table. Conservation of the *IL3* insulator CTCF sites in mammalian species.
Alignments of the conserved bases within the *IL3* +2.9 and +4.2 CTCF binding sites. The composite consensus sequence is shown above the sequence alignments. Identities are depicted as dots. Lower case letters within the alignment indicate an allowable base based on the consensus. Capital letters represent unfavourable sequence changes. Underlined segments represent conserved sequences.

S4 Table

***UPK2* gene intron CTCF site**

```
                            2° upstream         1° core
                               motif            motif
Global consensus            CTGCAGTTCCNNNTGGCCACCAGGGGGCGCC
of composite sites          G  TTACGTT   C     G   AT   AGT
                                 C

HUMAN             GGTGCAGCTTCTGCAGCCCAAGCCTGCCACCTGGTGGTCATACT
CHIMP             ...........................................C
GORILLA           ...........................................C
ORANGUTAN         ................................A...........
RHESUS            ...........................................C
BABOON            ...........................................C
MARMOSET          CA...........................T..........c.TG
TARSIER           T.......................................c.GC
MOUSE LEMUR       T.......................................c..G
BUSHBABY          T.....CT....-...........................c...
MOUSE             TACC..C.A........t...........T..........G.TC
RAT               TACC..C.A........t...........T............TC
KANGAROO RAT      TACCT........t........T.................G...
GUINEA PIG        C.GT.......A....tg..........C........c..G...
SQUIRREL          TA............G.........................GG..
RABBIT            CACC...T..........................C..G..G..C
PIKA              CA.C..............................C..G..G..A
DOLPHIN           CACCT............t.....................gc..A
COW               TACC.......................................G
HORSE             TA.C.G..................................G..A
CAT               .ACC.G....T...GT........................c..A
DOG               C..C....................................c..A
MICROBAT          TACC................C.............C....gG..C
MEGABAT           TA.C..................................TgG..C
HEDGEHOG          TA.T..............t.........C...........G..C
SHREW             .AC...........G...tG..G.....C........c..GG.C
ELEPHANT          TA.C.............................C...c..G..C
ROCK HYRAX        T.CC................................Ac..G..C
TENREC            TA.C....G.gG.....t.........................C
ARMADILLO         CCCC.G...............................c..G...
```

S4 Table. Conservation of the *UPK2* intronic CTCF site in mammalian species.
Alignments of the conserved bases within the *UPK2* CTCF binding sites. The composite consensus sequence is shown above the sequence alignments. Identities are depicted as dots. Lower case letters within the alignment indicate an allowable base based on the consensus. Capital letters represent unfavourable sequence changes. Underlined segments represent conserved sequences.

S5 Table

***HOXA9* chromatin boundary**

```
                              2° upstream         1° core
                                 motif            motif
Global consensus               CTGCAGTTCCNNNTGGCCACCAGGGGGCGCC
of composite sites             G  TTACGTT   C     G   AT   AGT
                                    C

HUMAN                   GCTGAGGCTGCAGTACCAAACGGCGGCCAGCAGATGGCAGTG
CHIMP                   ...................G......................
GORILLA                 ..........................................
ORANGUTAN               .......A.........G..TA.T..................
RHESUS                  ......A..........G..T.AT..................
SQUIRREL                T.G.G.........G.T..GGTAA......g...........
MOUSE                   ....C.........G....GAAAATT....g...........
RAT                     ....C.........Gtt..GAAAA.T....g...........
GUINEA PIG              ..C.G.........G.GCGCGC..A..............C..
RABBIT                  --------......Gt.C.GGCC...................
PIKA                    --------......G..C.G.AT...................
DOLPHIN                 ...TG-........Gt..GGGT...................A
COW                     ....G-........Gt...GGCA..................A
CAT                     ....G.........G..C.GATT..................A
DOG                     ..C.G.........G.AG.GAT....................
HEDGEHOG                ..G.G-A.......G..GGCAA.T.A....a...........
SHREW                   ..G.--.TC.....G.GGCG......................
ELEPHANT                --CTG-........G..TC.GT....................
TENREC                  --CTGC...........GC.GTTT..T..........G..CA
ARMADILLO               --G.GA........T..T.CG.AT..................
```

S5 Table. Conservation of the *HOXA9* chromatin boundary CTCF site in mammalian species. Alignments of the conserved bases within the *HOXA9* CTCF binding sites. The composite consensus sequence is shown above the sequence alignments. Identities are depicted as dots. Lower case letters within the alignment indicate an allowable base based on the consensus. Capital letters represent unfavourable sequence changes. Underlined segments represent conserved sequences.

S6 table   CTCF sites at histone H3 K27me3 chromatin boundaries

FoxJ3 Gene

>hg18_dna range=chr1:42411751-42411798
CGGCAGCC<u>CCGCCCCCTAGTGGCCACAGGCACTCACTACAG</u>CCTAATT

```
                       2° upstream          1° core
                          motif              motif
Global consensus       CTGCAGTTCCNNNNNNTGGCCACCAGGGGGCGCC
of composite sites     G  TTACGTT      C     G   AT   AGT
                            C
HUMAN           AATTAGGCTGTAGTGAGTGCCTGTGGCCACTAGGGGGCGGGGCTG
RHESUS          ...G......c......................a...T.......
TREE SHREW      .G.GG.....c......CA...A.......c..a.......AG..
MOUSE           .GGC......c.TG..A........AT...c.......a.cC...
RAT             GCAA......c.TG..A.........T...c.......accC...
GUINEA PIG      ..AG......ct................g.c..a....a.AT...
RABBIT          CG.G......c...A..CA......A....c..a....a......
HEDGEHOG        GC.C......c..-...CA.TA.......gc..a....a.A.GGC
DOG             .GCG......c......C.G.A.c....ggc..a.....c.TGGC
CAT             .GCG......c......CA..G.G......c..a....a.A.GGC
HORSE           .G.G......c......CA..G.A......c..a......A.GGC
COW             C..G......c...A..GAG.A.A....g.c..a.....cA.GGC
ARMADILLO       ...GT.....c......CA.....t.....c..a....a.A.GGC
ELEPHANT        ...G.A....c..CA..CA......A....c..a......A.GGC
TENREC          CG.G.A....c......CA...........c..a..Ct.AcTGCT
```

ABCG8 gene

>hg18_dna range=chr2:43958688-43958741
CAGGGTG<u>CTGCAGTGGCACAGACCAGCCACAGGATGGCAGT</u>AGAATAAAGACAGTCGAAAG

```
               2° upstream          1° core
                  motif              motif
Consensus      CTGCAGTTCCNNNNNNTGGCCACCAGGGGGCGCC
               G  TTACGTT      C     G   AT   AGT
                    C
HUMAN       GTGCTGCAGTGGCACAGACCAGCCACAGGATGGCAGTAGAATAAAGACAGTCGA
RHESUS      ......................................................
TREE SHREW  .C......c....C......T..............................T.G
MOUSE       .................GT................................T..
RAT         .................GT................................T..
GUINEA PIG  .................GT................................T..
RABBIT      .G..........t....GT.G..............................T..
DOG         A..T.........G...G.TG..............................A..
CAT         AATg.............G.TG..............................G..
HORSE       .G...A...........G..G................................G
COW         C................G.TG...............................A.
ELEPHANT    .G............G.AGT.G.....G........................A..
TENREC      CG......c.......C...G.............................AT..
```

S6 Table. Conservation of chromatin boundary CTCF sites in mammalian species.
Alignments of the conserved bases within the *FOXJ3* and *ABCG8* CTCF binding sites. The composite consensus sequence is shown above the sequence alignments. Identities are depicted as dots. Lower case letters within the alignment indicate an allowable base based on the consensus. Capital letters represent unfavourable sequence changes. Underlined segments represent conserved sequences. These are examples of CTCF sites that function to block the spread of repressed chromatin domains modified by Polycomb group proteins at developmentally regulated loci.

S7 table CTCF sites at histone H3 K9me2 chromatin boundaries

		2° upstream motif		1° core motif	References
Zinc fingers		11 10 9		7 6 5 4 3	[33]
Global CTCF motif		**CTGCAGTTCC**	nnn--nn	**TGGCCACCAGGGGGCGCC**	[41-43]
		G TTACGTT<br>C		C G AT AGT	
BIDC1	CCGCCCG	**CTGCACCGTG**	AGGCGCG	**CCAGGAGGAGGCGCAGGC**G	[49]
FMC1	TTTCCAG	**GAGGAGCTTT**	GCTCCAG	**CGGCCGTTAGATGGCGCC**T	[49]

BIDC1 CTCF site hg18 chr12:32,106,662-32,106,703
FMC1 CTCF site hg18 chr7:139,340,232-139,340,274

S7 table. CTCF sites at histone H3 K27me2 chromatin boundaries.
The above 2 CTCF sites from the *BICD1* and *FMC1* loci were identified by Shin et al [49] within regions representing chromatin boundaries separating active genes from repressed chromatin marked by the histone H3 K9me2 modification. These sites are likely to block the spread of heterochromatin. The coordinates of these chromatin barrier CTCF sites are shown below the alignments.
