# Supplementary material for: An upstream secondary DNA motif within the IL3 insulator CTCF binding site is required for enhancer-blocking insulator activity
Source: PLoS One. 2026 Feb 9;21(2):e0340247. doi: 10.1371/journal.pone.0340247 (PMC12885311; doi:10.1371/journal.pone.0340247)
Supplement: Attachment — Submitted filename: Response to CTCF paper Reviewer and Editor comments.docx [file pone.0340247.s003.docx]

**RESPONSES TO THE REVIEWERS**

Reviewer #1:

We thank reviewer 1 for their supportive comments.

There were no modifications requested by reviewer 1.

Reviewer #2: Summary

We also reviewer 2 for their supportive comments which are summarised here:

*The methods and results are scientifically sound, detailed, clearly presented and will be of interest to the transcriptional regulation, genome organization and developmental biology communities.*

We note that there were no substantial revisions requested, but just some minor comments.

As outlined below we have either (a) modified the paper according to the requests, (b) been unable to add new data, or (c) explained why additional data is not required.

*Minor comments and one experimental suggestion are provided below to enhance the paper.*

*Minor comments*
*1. Adding a more graphical representation to summarize the CTCF protein domain organization and previously characterized binding modes (of the ZFs) would enhance Figure 1.*

This was a very good suggestion. We have added a new figure 1 where we have drawn a model of CTCF binding to DNA based closely on the actual structural data from Yang et al. This appears in lines 80-95.

*2. The sub-section “The upstream CTCF motif is a common feature of enhancer insulators and chromatin barriers” should be moved to the beginning of the Results section.*

This was also a good suggestion. We have moved the description of known insulators to the beginning of the results. This required some juggling of text but the paper reads better now.

While doing this we also discovered that a new publication by Shin et al on CTCF sites chromatin barriers had been published in 2026 after we submitted this manuscript. This describes an additional major class of barrier that blocks spread of H3K9me2 which now adds to our previous description of barriers that block spread of H3K27me3. The new text appears in lines 188-196.

*3. A control should be added to the luciferase assay experiments described in lines 182-188.*

We disagree with the reviewer because the only control required is the pIL3 construct that contains the promoter alone. All the constructs are assayeded under the same conditions, so the data is already internally controlled. Each assay is also normalised to a Renilla luciferase cotransfected control. These constructs are inactive in the absence of stimulation, so there is no point in show nil values for unstimulated cells. Nevertheless, we added an extra sentence at lines 246-248 to the manuscript explaining that these constructs are inactive in the absence of stimulation and we cited out studies proving this point. There is no point repeating our published data.

*The CTCF site should also be tested in the forward orientation to complement the reverse orientation results. Based on the hypothesis presented, this should reduce the insulator activity and importantly would shed light on the functional role of the molecular organization of the protein-DNA complex.*

This is also a great suggestion but we do not have the resources to make additional constructs or perform new assays. This is beyond the scope of this study which aimed at suggesting future studies that other groups could perform. This was listed as a minor suggestion so we assume this was optional.

**RESPONSES TO THE EDITOR**

The editor suggested that we include a lab protocol for the studies described in this manuscript. However, we consider this to be not essential because all of the methods have been previously published by us and represent standard protocols used by most molecular biology labs. Furthermore, we do not have a single lab protocol but many linked protocols, and the effort to do this would be equal to writing a new manuscript.

This study was funded by the UK BBSRC.

Can the editor please include a funding statement in the appropriate place that reads:

This work was supported by Biotechnology and Biological Sciences Research Council grant BB/E023002/1 awarded to P.N.C. We declare that the funders had no role in study design, data collection and analysis, decision to publish, or preparation of the manuscript.

We note that the submission guidelines do not ask for a section called “Funding statement but just acknowledgements. The funding section of the submission form asked for funders but not the role of the funders as far as we could see.

In the acknowledgements we aalready state that.

This work was supported by Biotechnology and Biological Sciences Research Council grant BB/E023002/1 awarded to P.N.C.

If this is not appropriate then remove the acknowledgements section.

Yours faithfully

Peter Cockerill

University of Birmingham
